# Design and Development of Microscale Thickness Shear Mode (TSM) Resonators for Sensing Neuronal Adhesion

**DOI:** 10.3389/fnins.2019.00518

**Published:** 2019-06-04

**Authors:** Massoud L. Khraiche, Jonathan Rogul, Jit Muthuswamy

**Affiliations:** ^1^Neural Engineering and Nanobiosensors Group, Biomedical Engineering Program, Maroun Semaan Faculty of Engineering and Architecture, American University of Beirut, Beirut, Lebanon; ^2^Neural Microsystems Laboratory, School of Biological and Health Systems Engineering, Arizona State University (ASU), Tempe, AZ, United States

**Keywords:** quartz crystal microbalance (QCM), ultrasound, adhesion, neural interfaces, carbon nanotubes, microelectrode, neuron, acoustic sensors

## Abstract

The overall goal of this study is to develop thickness shear mode (TSM) resonators for the real-time, label-free, non-destructive sensing of biological adhesion events in small populations (hundreds) of neurons, in a cell culture medium and subsequently *in vivo* in the future. Such measurements will enable the discovery of the role of biomechanical events in neuronal function and dysfunction. Conventional TSM resonators have been used for chemical sensing and biosensing applications in media, with hundreds of thousands of cells in culture. However, the sensitivity and spatial resolution of conventional TSM devices need to be further enhanced for sensing smaller cell populations or molecules of interest. In this report, we focus on key challenges such as eliminating inharmonics in solution and maximizing *Q*-factor while simultaneously miniaturizing the active sensing (electrode) area to make them suitable for small populations of cells. We used theoretical expressions for sensitivity and electrode area of TSM sensors operating in liquid. As a validation of the above design effort, we fabricated prototype TSM sensors with resonant frequencies of 42, 47, 75, and 90 MHz and characterized their performance in liquid using electrode diameters of 150, 200, 400, 800, and 1,200 μm and electrode thicknesses of 33 and 230 nm. We validated a candidate TSM resonator with the highest sensitivity and *Q*-factor for real-time monitoring of the adhesion of cortical neurons. We reduced the size of the sensing area to 150–400 μm for TSM devices, improving the spatial resolution by monitoring few 100–1,000s of neurons. Finally, we modified the electrode surface with single-walled carbon nanotubes (SWCNT) to further enhance adhesion and sensitivity of the TSM sensor to adhering neurons (Marx, 2003).

## 1. Introduction

The biomechanics of the neuron-implant interface, involving highly localized neuronal adhesion, has a significant impact on intra- and extracellular signal fidelity, signal-to-noise ratio, and the viability of the neural tissue that determines the duration of neuronal recordings *in vitro* or life of an implanted device *in vivo*. Neurons change shape and realign their cytoskeleton to adhere to foreign substrates in their proximity. The complexity and dynamic nature of the adhesion process presents a challenge for studying this phenomenon using state-of-the-art end point imaging techniques such as fluorescent and electron microscopy. Electrical impedance-based methods lack the sensitivity required to capture changes in focal adhesion complexes (protein cytoskeletal anchor points at cell/substrate) since the measured current flows through the entire cell. Piezoelectric transducers have widely been used for sensing cellular adhesion due to their ease of use, low cost and high sensitivity. Quartz crystals are the most commonly used piezoelectric material for building transducers due to their desirable mechanical, thermal, chemical and electrical properties (Sauerbrey, 1959; Ferreira et al., 2009). AT-cut quartz produces bulk transverse shear waves with particle displacements parallel to the surface of the crystal and its electrodes. These AT-cut quartz oscillators are commonly termed thickness shear mode (TSM) resonators. When a small mass is deposited on the surface of a quartz crystal oscillator, the oscillator's resonance frequency decreases in direct proportion to the deposited mass as described by the classic Sauerbrey equation:

(1) $$\begin{array}{l} \Delta f_{o}=\frac{2f_{o}^{2}}{{\left(\rho_{Q}\mu_{Q}\right)}^{1/2}}\frac{\Delta m}{A} \end{array}$$

where *f*_*o*_ is the fundamental resonant frequency of the quartz crystal, A is the surface area of the piezoelectric area of the crystal, μ_*Q*_ and ρ_*Q*_ are the shear modulus and the density of quartz (Sauerbrey, 1959). The stability of AT-cut quartz under temperature change has led to a wide range of applications involving measurement of mass deposition in vacuum. TSM devices can operate in liquid and have been used for a variety of chemical or biosensor applications which include detection and analysis of proteins (serum, neurotransmitters) (Wang and Muthuswamy, 2008), antibodies as well as DNA (Ferreira et al., 2009; Li et al., 2011), self-assembled monolayer (SAMs) (Seker et al., 2016), lipids and cells (neurons, fibroblast, blood cells, neutrophils, bacteria) (Khraiche et al., 2003, 2005; Da-Silva et al., 2012; Khraiche and Muthuswamy, 2012; Zhou et al., 2012; Westas et al., 2015). The first attempts to study adhesion of cells using TSM sensors involved platelet adhesion (Matsuda, 1992). In the above report, Matsuda et al. concluded that the time dependent response of the acoustic sensor to cell attachment was not just due to the number of cells attaching but also to the adhesion state of the cells. In addition, work done by Gryte et al. (1993) showed that resonant frequency of the TSM sensor recovers to baseline when the pH was changed drastically causing the cells to detach from the sensor surface. Additionally, adding nonadherent beads to the sensor surface showed no change in resonant frequency. These early findings demonstrating the specificity of the changes in resonant frequency of the TSM sensors to cellular adhesion events opened the door to multiple studies using TSM sensors for monitoring adhesion (Khraiche et al., 2005; Khraiche and Muthuswamy, 2012; Lee et al., 2012; Saitakis and Gizeli, 2012; Da-Silva et al., 2013). Although the use of TSM resonators as biosensors encompasses a large number of applications, the commonly used dimensions of the electrodes and resonant frequencies of the crystal in most studies have a narrow range between 5 and 7 mm for electrode diameters, 200 nm for electrode thickness and 5–10 MHz resonant frequencies for the quartz crystal (Kosslinger et al., 1995). The above parameters of TSM resonators result in sensitivity (using Equation 1) and sensing area that is typically suitable for measurements of biological events in tens or hundreds of thousands of cells. Their performance therefore falls short of other comparable biosensing modalities such as surface plasmon resonance (SPR) (Su et al., 2005; Fang et al., 2015). In this study, we aim to develop a TSM sensor that can monitor biological adhesion in tens or hundreds of neurons *in vitro*. If successful, the natural advantages of TSM sensors such as label-free, non-destructive and real-time monitoring capabilities can be used to monitor the biomechanics of neurophysiology with much higher spatial resolution in a countable number of cells. Besides playing a key role in neuronal function and dysfunction in several pathologies, neuronal adhesion to a brain implant *in vivo* is also an important part of the immune response to the implants. Therefore, the proposed approach has potential *in vivo* applications in the future. The focus of this study is to investigate the effect of resonant frequency of TSM sensors and electrode dimensions (active sensing area) on sensitivity and *Q*-factor in the context of sensing neuronal adhesion. As indicated by Equation (1), the sensitivity of TSM sensors operating in air or vacuum is directly proportional to the square of the resonant frequency. But the fundamental resonant frequency in the bulk of the TSM sensor is limited by the thickness (wavelength of the fundamental is twice the thickness as indicated in Equation 2) of the quartz substrate. For instance, to fabricate a TSM sensor with 100 MHz resonant frequency, the thickness of the substrate needs to be approximately 16 μm which pose challenges to fabrication, handling and packaging. Furthermore, for sensing neuronal adhesion in biological media, two critical aspects of TSM sensor operation-inharmonic modes and the quality factor (*Q*-factor) need to be optimized. Inharmonic modes or spurious modes are standing waves in the quartz substrate with frequencies different from the resonant frequency and its harmonics. These unwanted inharmonics reduce acoustic energy trapping and, if the frequency separation between the harmonic and inharmonic modes is not sufficient, adjacent resonant peaks interfere with each other, resulting in mode coupling or frequency jumps. The *Q*-factor is considered a critical metric for sensor sensitivity as it determines the minimal frequency change detectable and is affected by dielectric, acoustic and electric losses within the TSM sensor. In this study, we specifically minimize inharmonic modes and maximize *Q*-factor and develop a design framework that serves as a guide for increasing sensitivity and spatial resolution of TSM sensors operating in a liquid environment.

## 2. Theory

### 2.1. Harmonic and Inharmonic Waves

The application of an electric field across the thickness of AT-cut quartz leads to particle movement, which in turn results in two types of standing waves-a transverse wave in the thickness direction referred to as the thickness shear TS1 wave and a wave traveling in the radial direction known as the thickness twist TT3 wave. The path length of TS1 waves is the quartz plate thickness with nodes along the diameter of the plate, while the path length for TT3 is the electrode radius with concentric nodal lines along the center of the quartz plate. When the length of the path is an integral number of wavelengths, a standing wave occurs and results in resonance (Shockley et al., 1967). The fundamental resonant frequency of AT-cut quartz is a result of the TS1 standing wave and is the most reliable and largest wave of this type of acoustic systems. TT3 waves are considered unwanted inharmonic waves that, if present, can interfere with the correct measurements of the resonant frequency.

### 2.2. Energy Trapping

The theory of energy trapping is based on the principle that waves traveling in a piezoelectric substrate must have a frequency higher than a certain “cutoff frequency,” defined as the fundamental frequency of the system, which is the frequency predicted based on the thickness of the quartz substrate (hs).

(2) $$\begin{array}{l} f_{0}=\frac{\sqrt{\mu_{Q}/\rho_{Q}}}{2h_{s}} \end{array}$$

where *f*_*o*_ is the fundamental resonant frequency of the quartz crystal, *A* is the surface area of the piezoelectric area of the crystal, μ_*Q*_ and ρ_*Q*_ are the shear modulus and the density of quartz (Ferreira et al., 2009).

**Figure 1:**
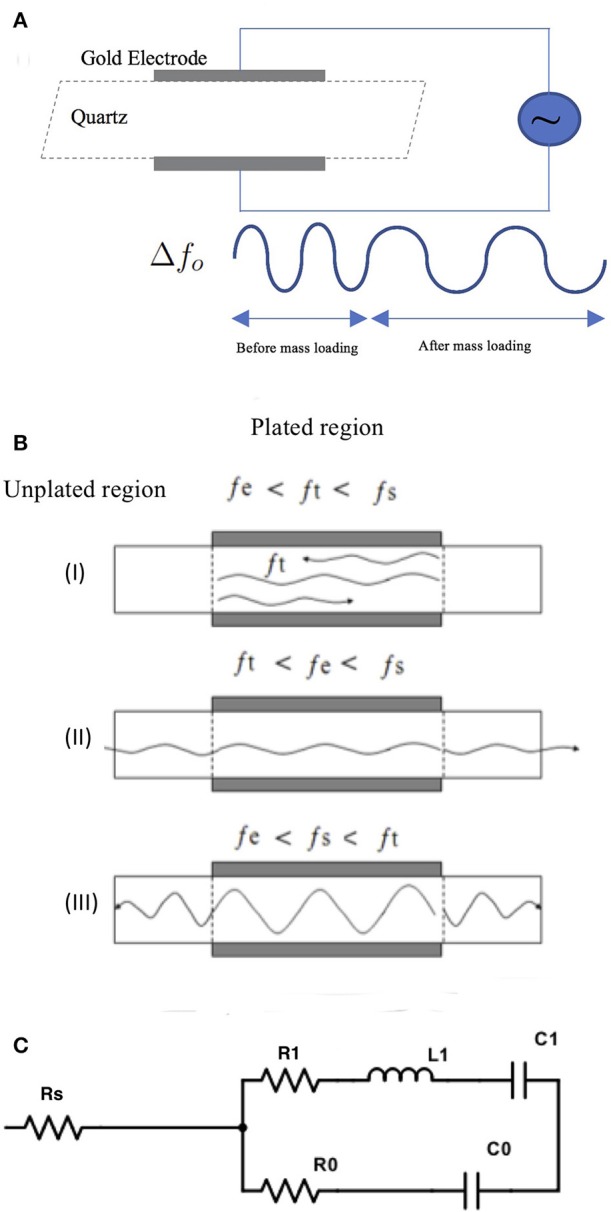
**(A)** Illustration of theory of operation of TSM devices. **(B)** Shows three scenarios for propagation of waves within the quartz crystal in a TSM sensor. For illustration, the waves are shown in a cross-section of the TSM sensor, where metalized electrodes over the quartz create two distinct regions with different cut-off frequencies for the propagated waveforms -unplated region “s” with a cutoff frequency *f*_*s*_ and plated region “e” with a cutoff frequency *f*_*e*_ with *f*_*s*_ > *f*_*e*_ due to the weight of the electrode. This gives rise to three different scenarios for propagation of waves of frequency *f*_*t*_ in the plated quartz substrate. (I) when *f*_*t*_ is between *f*_*e*_ and *f*_*s*_, waves can propagate within the “e” region but not into the “s” region, and total internal reflection occurs at the boundary between the two regions (II) when *f*_*t*_ is below *f*_*e*_, waves cannot propagate into “s” region but get attenuated within the “e” region III) when *f*_*t*_ is higher than *f*_*s*_, vibration energy generated in region “e” will propagate into the “s” region, resulting in loss of energy and therefore will not contribute to a localized standing wave response. **(C)** The BVD electrical model consists of three series components modified by the mass and viscous loading of the crystal where R1 is the dissipation of the oscillation, C1 corresponds to the stored energy in the oscillation, L1 (inductor) corresponds to the inertial component of the oscillation. The BVD model can be modified to a five-element model, in which a series resistor (RS) is added.

When electrodes are applied to an AT-cut quartz plate they create two regions of different frequencies as illustrated in Figure 1A (plated and unplated) where a cross section of the TSM sensor is shown. The two regions are formed due to the mass of the electrodes on the plated region “e.” resulting in the cutoff frequency (*f*_*s*_) of the unplated “s” region being slightly higher than the cutoff frequency (*f*_*e*_) of the plated region “e.” These two regions give rise to three different scenarios for the propagation of a wave (frequency *f*_*t*_) within the quartz. For waves having frequencies *f*_*t*_ where *f*_*e*_ <*f*_*t*_ <*f*_*s*_, these waves can propagate within the “e” region but not the “s” region, and total internal reflection occurs at the boundary between the two regions. Waves in the plated “e” region with frequencies below *f*_*e*_ (i.e., *f*_*t*_ <*f*_*e*_) cannot propagate in the plated region, “e” or into the unplated region, “s” and get attenuated within the plated “e” region. For waves with frequency, *f*_*t*_ higher than *f*_*s*_ (*f*_*t*_ >*f*_*s*_) vibration energy generated in plated region “e” will propagate away resulting in a loss of energy and, therefore, will not contribute to a localized standing wave response. The latter is desirable for unwanted waves.

### 2.3. Eliminating Inharmonic Waves

Based on the energy trapping theory, the frequency of the inharmonic waves needs to be higher than the cut-off frequency of the unplated region in order to eliminate the inharmonic modes. This will cause the inharmonic to travel in the unplated region without creating a standing wave in the crystal (Bottom, 1982). The eigen frequency of a clamped resonator *f*_*nmk*_ with a circular electrode can be calculated from the following equation;

(3) $$\begin{array}{l} f_{nmk}=f_{n01}\left(1+2\frac{X_{mk}h_{s}}{n\pi d}\right) \end{array}$$

*X*_*mk*_ is the *k*th root of the Bessel function of order *m*, *f*_*n*01_- the frequency of the *nth* harmonic mode, *d* is the diameter of the electrodes and hs is the thickness of the quartz. As mentioned earlier, if the first inharmonic is larger than the cut-off frequency of the unplated region, the inharmonic will propagate into the unplated region and will not result in a standing wave. So, our design should consider the following;

$$nf_{0}<f_{nmk}$$

where *f*_0_ is the fundamental resonant frequency. Given that *X*_11_=3.832 and substituting Equation (3) we have;

(4) $$\begin{array}{l} \Delta f_{pb}<f_{n01}\frac{2.98}{n^{2}}{\left(\frac{h_{s}}{d}\right)}^{2} \end{array}$$

We refer to Δ*f* as the plate-back. From Equation (4), we find a design guide for eliminating the inharmonic modes. We assume that the change in mass by adding electrodes is equivalent to the plate-back, which will allow us to equate plate-back to the Sauerbrey equation and substituting n=1 (first inharmonic), we get the following equation;

(5) $$\begin{array}{l} h_{e}<\frac{9.38X10^{8}m^{3}s^{-3}}{f_{0}^{3}d^{2}} \end{array}$$

Where the term *h*_*e*_ is thickness of the electroded region of the crystal (electrode + quartz). This equation provides a guide to designing a resonator while avoiding all inharmonic modes.

### 2.4. Liquid Load

However, when the sensor is operated under liquid loading, Equation (4) becomes inaccurate in predicting the presence of the inharmonics, due to the impact of viscoelastic loading on the QCM sensor under liquid load. Kanazawa et al used the velocity distribution of the crystal oscillation in fluid to describe how the viscosity and density of the fluid affects the oscillation. The result of that work was an expression describing the frequency change induced by immersing one face of a quartz resonator in a liquid as a function of the viscosity as well as the density of the liquid (Kanazawa and Gordon, 1985).

(6) $$\begin{array}{l} \Delta \text{f}=-{\text{f}}_{0}^{\frac{3}{2}}{\left(\frac{\rho_{l}\eta_{l}}{\pi \rho_{q}\mu_{q}}\right)}^{\frac{1}{2}} \end{array}$$

where η_*l*_ is the viscosity of the liquid and ρ_*l*_ is the liquid's density, ρ_*q*_ and μ_*q*_ are the density and shear modulus of the quartz. The net change in resonant frequency is therefore the summation of the mass loading effects described by Sauerbrey and the viscosity effects described by Kanazawa's equations. If we assume that plate-back is equivalent to the change in frequency from both of these mass loadings, we have

(7) $$\begin{array}{l} \Delta \text{f}=\frac{-2{\text{f}}_{0}^{2}\Delta m}{v_{q}\rho_{q}\text{A}}-{\text{f}}_{0}^{\frac{3}{2}}\sqrt{\frac{\eta_{l}\rho_{l}}{\pi \mu_{q}\rho_{q}}} \end{array}$$

And

(8) $$\begin{array}{l} \Delta \text{f}=\frac{-2{\text{f}}_{0}^{2}\Delta m}{v_{q}\rho_{q}\text{A}}-{\text{f}}_{0}^{\frac{3}{2}}\sqrt{\frac{\eta_{l}\rho_{l}}{\pi \mu_{q}\rho_{q}}}<{\text{f}}_{n01}\frac{2.98}{n^{2}}{\left(\frac{{\text{h}}_{s}}{d}\right)}^{2} \end{array}$$

The equation can be rearranged to;

(9) $$\begin{array}{l} {\text{h}}_{e}<\frac{2.98\mu_{q}v_{q}}{16n^{2}{\text{f}}_{0}^{3}d^{2}\rho_{e}}-{\text{f}}_{0}^{\frac{3}{2}}\sqrt{\frac{\eta_{l}\rho_{l}}{\pi \mu_{q}\rho_{q}}}\frac{v_{q}\rho_{q}}{4{\text{f}}_{0}^{2}\rho_{e}} \end{array}$$

(10) $$\begin{array}{l} {\text{h}}_{e}<\frac{2.98\mu_{q}v_{q}}{16n^{2}{\text{f}}_{0}^{3}d^{2}\rho_{e}}-\sqrt{\frac{\eta_{l}\rho_{l}\rho_{q}}{\pi \mu_{q}{\text{f}}_{0}}}\frac{v_{q}}{4\rho_{e}} \end{array}$$

Assuming that the liquid is water at 20^*o*^C with a viscosity of 1.0022 × 10^−3^ Pa.s [or kg/(m.s)] and a density of 998.2 kg/*m*^3^, then the equation further reduces to:

(11) $$\begin{array}{l} {\text{h}}_{e}<\frac{9.38X10^{8}m^{3}s^{-3}}{{\text{f}}^{3}d^{2}}-\sqrt{\frac{5.32X10^{11}m^{2}s^{-1}}{{\text{f}}_{0}}} \end{array}$$

### 2.5. *Q*-Factor

The quality factor (*Q*-factor) for resonators is the ratio between energy stored and the energy dissipated per cycle. This quantity is a metric for sensor efficiency and stability and can be calculated based on material properties. The following equation relates the *Q*-factor to the resonant frequency of the quartz (Mason, 1956);

(12) $$\begin{array}{l} \text{Q}=1.6X10^{13}\frac{1}{{\text{f}}_{0}} \end{array}$$

The *Q*-factor value calculated in Equation (12) is not reached in experimental prototypes. This is due to two types of losses in TSM oscillators-electrical losses and acoustic losses. The electrical losses stem from the electrical properties of the electrodes sandwiching the quartz crystal in the TSM sensor and the associated leads. As for the acoustic losses, it includes internal losses due to defects, scattering and losses due to acoustic boundaries between thin films in the oscillator. In addition, for TSM sensors operating in fluid, the acoustic energy is dissipated in the fluid by viscous mechanisms resulting in a decrease in the quality factor. While a thin solid film will only cause a change in resonant frequency, a Newtonian liquid will cause a simultaneous shift in the resonant frequency and the decrease of the quality factor. That being said, a more accurate estimate of the *Q*-factor can be obtained from the BVD (Butterworth van dyke) model. The classic BVD model consists of a series of *R*_1_, *L*_1_, and *C*_1_ components modified by the mass and viscous loading of the crystal where *R*_1_ is the dissipation of the oscillation, *C*_1_ corresponds to the stored energy in the oscillation, *L*_1_ corresponds to the inertial component of the oscillation. The BVD model can be further modified to better describe the crystal, and the losses impacting the *Q*-factor, as a six-element model, in which a series resistor (*R*_*S*_) and acoustic leakage resistance Ro is added (Figure 1C). The addition of RS accounts for losses due to the electrode electrical properties. Past work has shown that this modified BVD model improves *Q*-factor estimates and relates electrode conductivity to *Q*-factor changes (Larson et al., 2000). In addition, the *Q*-factor's impact on TSM sensor sensitivity can be determined by considering the smallest detectable resonant frequency, which is governed largely by the *Q*-factor and can be calculated from the following (Lakin et al., 1993; Weber et al., 2008);

(13) $$\begin{array}{l} \Delta f_{o}=\frac{1}{2}f_{o}\frac{\Delta \phi_{min}}{Q} \end{array}$$

The previous equation relates the smallest detectable change in the resonant frequency by TSM sensors to the *Q*-factor and ϕ (phase resolution of the acquisition system). Using Equation (1) we can rewrite Equation (13) for sensitivity in terms of the smallest change in mass per unit area:

(14) $$\begin{array}{l} \frac{\Delta m_{min}}{A}=\frac{1}{2}\frac{\sqrt{\rho_{Q}\mu_{Q}}\Delta \phi_{min}}{f_{o}Q} \end{array}$$

### 2.6. Motional Resistance and Viscoelastic Changes

Through an electromechanical analogy of the quartz crystal, Muramatsu et al. derived a relationship between the motional resistance *R*_1_ and the density and viscosity of the liquid that is shown below in Equation (15):

(15) $$\begin{array}{l} {\text{R}}_{1}=\frac{{\left(2\pi {\text{f}}_{0}\rho_{l}\eta_{l}\right)}^{\frac{1}{2}}A}{\kappa^{2}} \end{array}$$

Where *A* is the active electrode area of the sensor, ρ_*l*_ is the density and η_*L*_ is the absolute viscosity of the liquid, κ an electro-mechanical coupling factor (Kanazawa and Gordon, 1985; Muramatsu and Kimura, 1992). A plot of Δ*R*_1_ vs. Δ*f*_*s*_ has then been used to describe the mechanical changes in the deposited thin film on the sensor surface (Zhou and Muthuswamy, 2004). The term (ρ_*L*_η_*L*_) appears in both Equations (6) and (14) and the ratio of both equations resulting in a straight line in the case of pure viscous liquid contacting the sensor surface. The relationship of Δ*R*_1_ vs. Δ*f*_*s*_ provides insights into viscoelastic changes in the thin layer adhering to the sensor, where changes in frequency only (without a corresponding change in Δ*R*_1_), are due to rigid mass deposition (elastic changes) and changes in viscosity of the adhering layer only, will result in changes along a line of unit slope in the Δ*R*_1_ vs. Δ*f*_*s*_ plot (energy dissipation). This type of plot was introduced by Muramatsu et al (Muramatsu and Kimura, 1992; Kang et al., 2008) and was later used by Marx et al in studying thin film deposition and cells (Marx, 2003).

## 3. Materials and Methods

### 3.1. Sensor Fabrication

**Figure 2:**
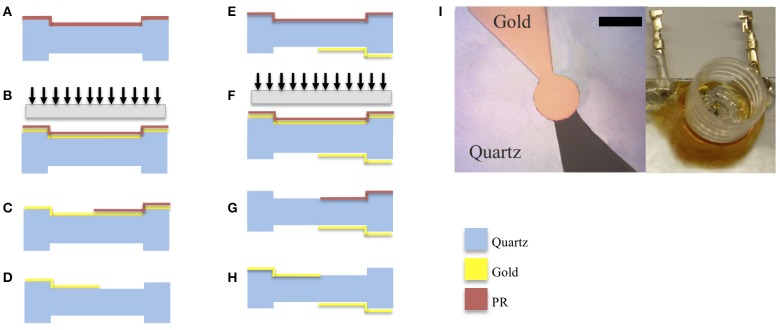
Schematic of the fabrication process involving deposition of gold electrodes on quartz substrates with inverted mesa-like out-of-plane structures. Figures on the left-hand column correspond to the patterning of the topside of the quartz plate. **(A)** gold was vapor deposited on the quartz surface. **(B)** photoresist (PR) patterned on gold and pattern exposed. **(C)** solvent used to wash away exposed PR. **(D)** gold etchant used to expose electrode. Figures on the right-hand column correspond to patterning of the bottom side of the quartz plate. The quartz wafer was flipped to expose the bottom side, **(E)** photoresist coated on quartz **(F)** photoresist (PR) patterned on gold and pattern exposed using backside alignment. **(G)** solvent used to wash away exposed PR; **(H)** gold is evaporated to form the electrode on the bottom side. **(I)** Micrograph of the resulting pattern of gold electrodes on 50 Hz quartz (scale bar 150 μm) along with a picture of the cell culture well toping the crystal with interconnects.

High frequency polished quartz crystals were obtained from Xeco, Cedar City, UT, USA. The crystals had an inverted mesa structure that provided a thick outer frame for handling while having a thin inner membrane. Photoresist was used to adhere the quartz to glass slides that were used as chips carrier. A metallization layer of chrome/gold was then deposited by thermal evaporation as illustrated in Figure 2. PR (AZ4620) was spun coated on the crystal without the ramp step to reduce beading on the crystal edge. Using a photomask, the edge beading was removed from the crystal by overexposing the edges and developing it in 400T developer for 2 min at 1:3 concentrations. The pattern of the first side was exposed using a laser aligner (EVG620) at 700 mJ/*cm*^2^ and developed in 300 MIF for 2 min. Gold etchant was used to etch away the exposed gold, revealing the pattern on the crystals. The quartz wafers were then removed from the glass slides using acetone, cleaned and remounted with the new pattern facing down. A layer of photoresist (PR) AZ4620 was spin-coated on the crystal at 5,000 rpm without a ramp-up step and the crystal was baked at 100^*o*^C for 2 min. The pattern of the second side was exposed after being aligned with the bottom side using a laser aligner (EVG620) at 700 mJ/*cm*^2^ and was developed in 300 MIF for 2 min. Gold was then deposited on the exposed areas. The next step involved washing away the gold that was deposited on the PR and exposing the top pattern resulting in overlapped electrodes on each side of the crystal (Figure 2I).

### 3.2. Device Cleaning and Post-processing

To remove organic deposits from the surface of the devices, the quartz crystal and gold electrode were cleaned using piranha solution (*H*_2_*O*_2_ + *H*_2_*SO*_4_) 3:1 v/v) for 5 min, then rinsed with DI (de-ionized water) water and dried in air for 15 min. The crystal was then treated with 1 M NaOH for 20 min, then rinsed with DI water (Khraiche and Muthuswamy, 2012).

### 3.3. Surface Modification Using Carbon Nanotubes (CNTs) to Enhance Sensitivity

Single walled carbon nano tubes have excellent properties for sensing including high conductivity, high surface to volume ratio which results in high surface roughness on the nanoscale [31–33]. The increase surface roughness of TSM electrodes has been shown to increase the absolute change in resonant frequency and subsequent sensitivity of TSM sensors (Daikhin and Michael Urbakh, 1997; Du and Johannsmann, 2004; Rechendorff et al., 2007). This increase in Δ*f* is due to interfacial slip between the solid and liquid phase, where there is an abrupt change in velocities at the interface. Several attempts have been made to understand this phenomenon and a model was developed to predict the impact on Δ*f* by deriving a term that can be added to the Kanazawa's expressions listed earlier (where no slip condition is assumed) (Equation 6):

(16) $$\begin{array}{l} \frac{\Delta f}{f^{2}\rho }=-\frac{1}{\sqrt{\rho_{0}\mu_{0}}}\left(\pi^{1/2}\frac{w^{2}}{\xi }+R\delta \right) \end{array}$$

Where *w* is the surface roughness, ξ is the correlation length of the roughness, R is a roughness factor and δ decay length. When surface roughness is equal to zero (smooth surface), then Equation (16) reduces to Equation (6). Single walled carbon nano tubes (SWNT) were purchased from Sigma Aldrich. The procedure of depositing SWNT was as previously reported (Gabriel et al., 2009). The SWNT were made into a black suspension by mixing 10 mg of pure SWNT with 10 ml of dimethyl formamide (DMF). The solution was placed under ultrasonic agitation for 50 min. Several drops were placed on the microelectrodes typically 5-10 μl and allowed to evaporate at 100^*o*^C. After the solution evaporated, the MEA was rinsed with DI water and wiped with clean room wipes. The steps were repeated 4–5 times until the microelectrodes appeared black. After mechanical polishing the carbon nanotubes (CNT) stayed only on the gold microelectrodes.

### 3.4. Data Acquisition

A network analyzer HP E5100A (Agilent Technologies, USA) connected to a PC was used to acquire and record the impedance spectrum via a custom LabVIEW^TM^ program. The network analyzer was equipped with a passive π-network fixture where the leads to the crystals were directly connected. Admittance spectrum was collected with 201 points around the center resonant frequency, at 200 KHz bandwidth and 0.5 dBm with an incident power of 1 mW.

### 3.5. Cell Culture

Neurons were extracted from cortical slices from embryonic day-18 Sprague-Dawley rats obtained from BrainBits (Springfield, IL). Slices arrived in Hibernate-E media. Mechanical titration was used to break down the slices, and the supernatant was spun for 1 min at 1,100 rpm and suspended in ActiveNB neuron growth media with B27, 25 μM glutamic acid and 0.5 mM l-glutamine. Cells were added in suspension to a sensor surface in 20 μl and left for 20 min to adhere before adding more growth media and before the start of the resonant frequency measurements. Prior to neuron seeding, the resonator surface was coated with laminin (20 ml of 1 mg/ml laminin diluted in 1 ml of growth medium) and PEI (.1% in borate buffer). Fabricated TSM devices were topped with wells that hold 1.5 ml of media (Figure 2, top right) and sealed with a fluorinated ethylene-propylene (FEP Teflon® film), a transparent, oxygen permeable and water impermeable membrane. The whole setup was then placed in a cell culture incubator (controlled CO2 and 98% humidity) for the duration of the experiment, and media levels were monitored constantly. After 7 days in culture, neural cell culture viability was checked using live/dead fluorescence assay and by measuring single unit activity. All experiments were conducted according to standard biosecurity and institutional safety procedures of Arizona State University, Tempe, AZ.

### 3.6. SEM Protocol

Growth media was rinsed off with PBS wash (three times). Samples were then fixed in 2.5% glutaraldehyde in 0.1 M cacodylate buffer at pH = 7.4 and left for a 1 h at room temperature. This was followed by a wash in PBS (three times) and the sample was left in PBS for 5 min after each rinse. The next step included adding PBS solution containing 1% osmium tetroxide to the sample for 1 h at room temperature to improve the contrast in imaging, followed by washing in distilled water (three times). Dehydration: The samples underwent a serial dehydration in 30, 50, 70, and 90% (10 min. each) and three times with 100% ethanol (within 15 min). Critical point drying: Samples were placed in a critical point dryer for 10–15 min and imaged afterward (Liu et al., 2017).

## 4. Results and Discussion

### 4.1. Plate-Back

**Figure 3:**
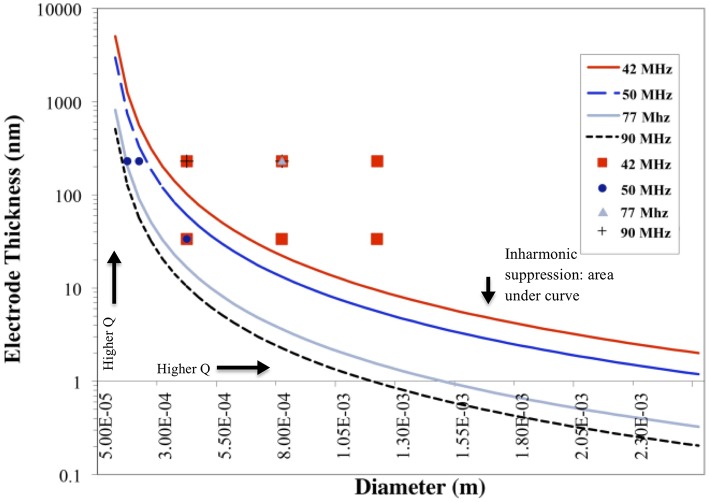
Plate-back characteristics for suppressing the unwanted inharmonic modes in air demonstrate the theoretical relation between electrode thickness and electrode diameter for any given resonant frequency (inversely proportional to the thickness of the quartz crystal). The lines correspond to the specific resonant frequency of the quartz crystal. The region under each curve indicates possible combinations of electrode thicknesses and diameters that will suppress inharmonic modes of vibration.

**Figure 4:**
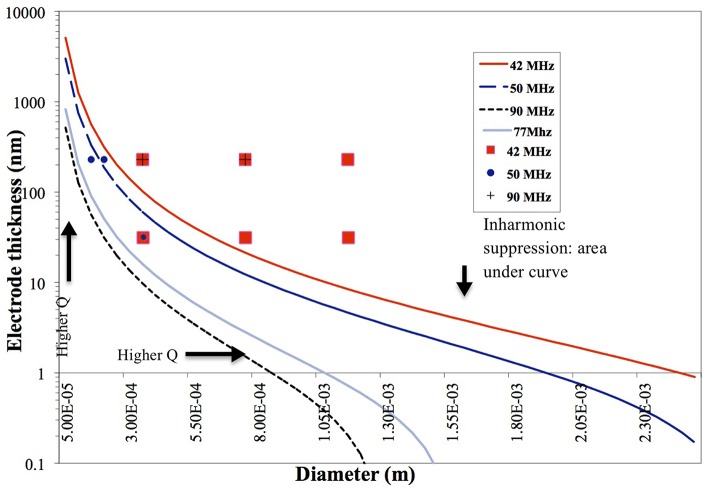
Plate-back characteristics for suppressing the unwanted inharmonic modes in liquid operation demonstrate the theoretical relation between electrode thickness and electrode diameter for any given resonant frequency (inversely proportional to the thickness of the quartz crystal). The region under each curve (corresponding to a specific resonant frequency of the quartz crystal) indicates possible combinations of electrode thicknesses and diameters that will suppress inharmonic modes of vibration.

**Figure 5:**
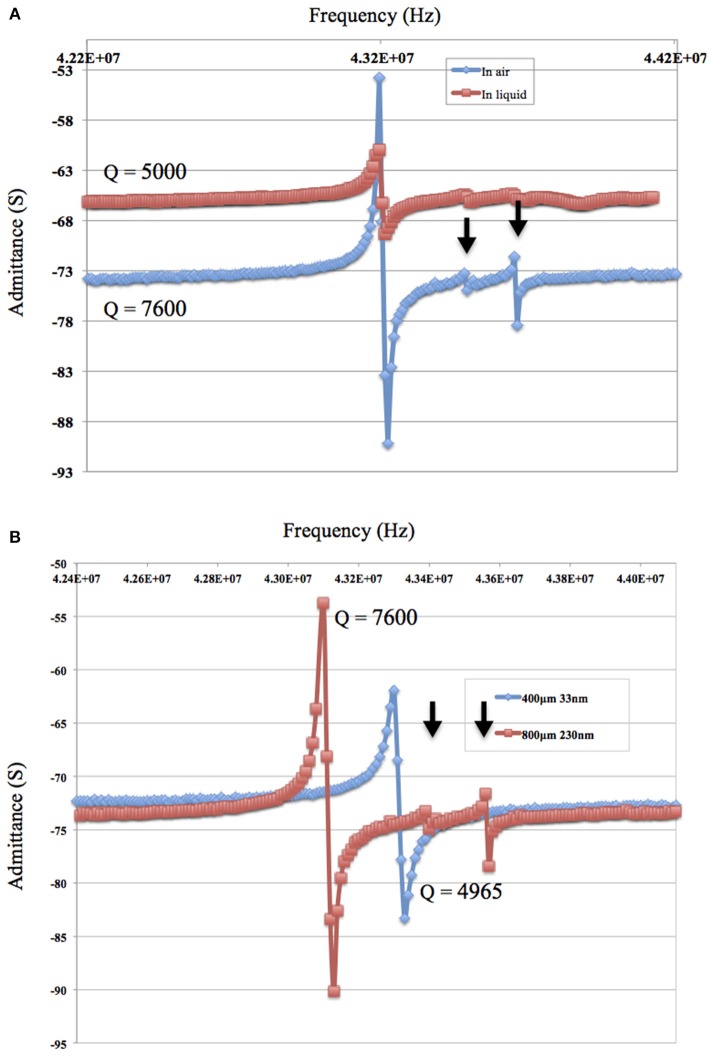
**(A)** The admittance spectrum of the 42 MHz TSM resonator in air and in phosphate buffered saline (PBS) show that inharmonics are more suppressed under liquid due to the liquid loading. The admittance of the TSM sensor at the most significant inharmonic is 1/6th the impedance at resonant frequency in air vs. 1/30th in PBS. **(B)** The admittance spectrum in air of 42 MHz and a gold electrode of 800 μm diameter and a thickness of 230 μm (in red) and the admittance spectrum of 42 MHz and a gold electrode of 400 μm diameter and a thickness of 33 nm (in blue).

Results plotted in Figure 3 represent the theoretical plate-back characteristics relating electrode thicknesses and electrode diameters for TSM resonators operating in air. Traces corresponding to 42, 50, 75 and 90 MHz are shown in Figure 3 along with points that correspond to the quartz TSM prototypes fabricated and tested in this study. The plate-back characteristics for suppressing the unwanted inharmonic modes change between operations in liquid (Figure 4) vs. air (Figure 3). Changes in admittance spectrum in response to TSM operation in air vs. liquid are shown in Figure 5A. Comparing the performance of the TSM resonator in air vs. liquid in Figure 5A, the inharmonics are more suppressed under liquid operation due to liquid loading. The admittance at the most significant inharmonic is 1/6th the admittance at the resonant frequency in air, compared to 1/30th in liquid. The usefulness of Figure 3 can be enhanced for biosensor design when we account for frequency changes due to the dampening effects of fluid loading or even target analyte (cells proteins). By incorporating Kanazawa's equation, that accounts for the density and viscosity of water at 20^*o*^C in the plate-back equations for eliminating inharmonics (Equation 10), Figure 4 is obtained as a guide for sensor design. When comparing Figure 3 with Figure 4, we found notable differences in the design space for suppressing inharmonics in TSM sensors operating in air vs. TSM sensors operating in liquid, for larger diameter electrodes. This relationship was based on the density and viscosity of water. This is especially evident for higher frequency resonators. If the sensor is to operate in a different medium, necessary changes to Equation 10 need to be made (Kanazawa and Gordon, 1985; Kanazawa, 2002). In order to suppress unwanted inharmonic modes based on the relationship defined by Equation 11, the region under the curve represents ideal resonator designs where the frequency of the first inharmonic is larger than the cut-off frequency of the unplated region of the sensor resulting in inharmonic suppression (as predicted by the plate-back Equation 11). An example of this is the admittance spectrum plots in Figure 5B of two 42 MHz sensors, one with a 400 μm diameter electrode and 33 nm of gold thickness (blue trace in Figure 5B) that corresponds to a point below the plate-back characteristics of 42 MHz sensor in Figure 4, while the second 42 MHz TSM sensor with 800 μm diameter electrode and 230 nm of gold thickness (red trace in Figure 5B) lies above the plate-back characteristics in Figure 4. Inharmonic frequencies are only observed in the trace corresponding to the second TSM sensor (in red; inharmonic modes indicated by arrows) and not the first. This result is consistent with Equation (11), where increasing the electrode thickness will increase the left side of the inequality resulting in more inharmonics.

### 4.2. Increasing Electrode Thickness

**Figure 6:**
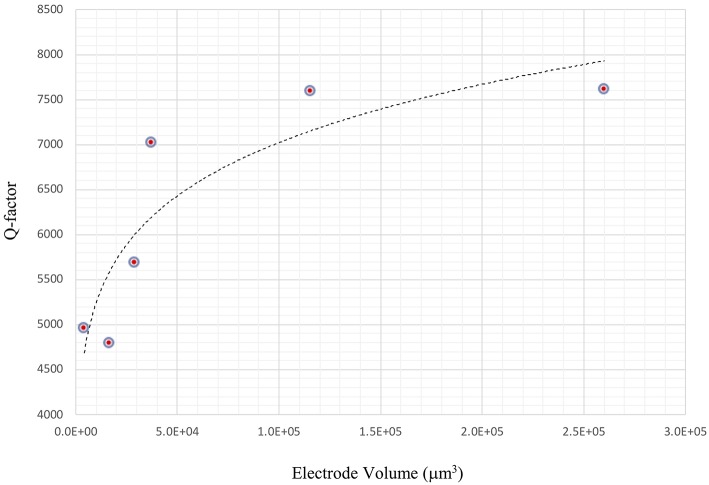
Improvement in *Q*-factor with increasing electrode thickness. Increasing electrode thickness increases the conductivity of the electrodes and reduces the resistance, which results in a higher *Q*-factor.

The impact of electrode thickness on TSM sensor performance is not immediately apparent. The impedance spectra of two TSM sensors in Figure 5A (in air) and Figure 5B (in water) reveal a higher *Q*-factor for the TSM sensor with 230 nm of gold thickness compared to the sensor with 33 nm of gold thickness. Changes in the thickness of deposited gold electrodes lead to changes in the electrical resistance. The dependence of the *Q*-factor of TSM devices on the resistivity can be better understood from the modified BVD model of a resonator. Adding a resistor in series with the BVD model for a bulk acoustic resonator accounts for changes in *Q*-factor and improves the accuracy of the model. Increasing electrode thickness increases the conductivity of the electrodes and reduces the resistance, which results in a higher *Q*-factor as shown also in Figure 6 as the *Q*-factor increases with increasing electrode volume.

### 4.3. Sensor Frequency

**Figure 7:**
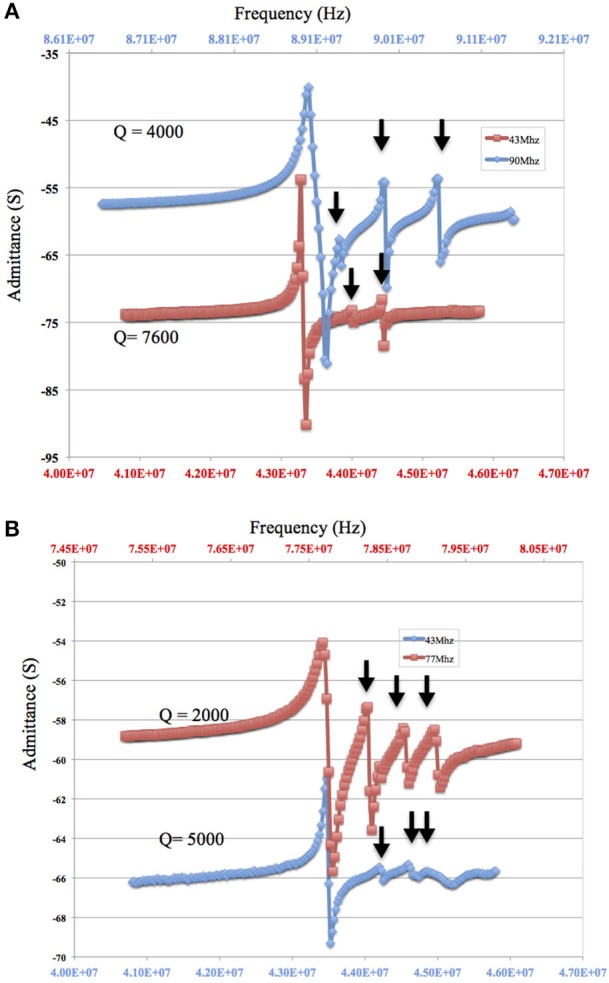
**(A,B)** Decreasing *Q*-factor and larger inharmonic modes with increasing resonant frequency of the quartz substrate. The admittance spectrum of two microresonators with a resonant frequency of approximately 90 MHz and an electrode of 800 μm diameter and a thickness of 33 nm (in blue) and a second with a resonant frequency of approximately 42 MHz and a gold electrode of 800 μm diameter and a thickness of 33 nm (in red) are shown (**A**: in air and **B**: in fluid with 90 MHz resonator replaced by a 77 MHz resonator). Arrow points to the inharmonic.

When considering sensor design, sensitivity requirements determine the choice of the fundamental resonant frequency of the TSM sensor and ultimately the choice of the thickness of the quartz. Increasing the resonant frequency of TSM sensors increases sensor sensitivity as shown by the Sauerbrey equation (Equation 1). On the other hand, increasing the resonant frequency of the TSM sensor lowers the *Q*-factor (Equation 12). This relationship is evident in the impedance sweep in Figure 7A showing two TSM prototypes with different resonant frequencies (90 MHz shown in blue and 42 MHz shown in red) but with the same electrode dimensions (800 μm diameter and 33 nm thickness of gold). The *Q*-factor for the TSM sensor with a resonant frequency of 42 MHz is 7,000 compared to 4,000 for the one with a resonant frequency of 90 MHz. The impact of frequency on the *Q*-factor has implications when operating in liquid, as the quartz undergoes hydrostatic dampening and dampening due to the viscous medium, both of which lead to a further reduction in the *Q*-factor (Figure 7B).

As for the impact of sensor fundamental resonant frequency on the inharmonic frequencies, plate-back characteristics in Figures 3, 4 indicate that the higher the resonant frequency of the quartz, the lower the inharmonic suppression, which has an adverse impact on the operation of the crystal. In Figure 7A, the first inharmonic appears to be larger and closer to the resonant frequency for the TSM sensor with a resonant frequency of 90 MHz compared to the resonant frequency of the 42 MHz resonator. Referring back to Figure 3, the point corresponding to the 90 MHz micro-resonator with its electrode diameter and thickness shown in Figure 7, lies farther above the plate-back characteristics of 90 MHz in comparison with the point corresponding to the 42 MHz micro-resonator, relative to its corresponding curve in Figure 3. Therefore, the 90 MHz micro-resonator profiled in Figure 7 is expected to generate significantly larger inharmonic modes than the 42 MHz resonator, and the results shown in Figure 7A confirms the presence of these modes.

### 4.4. Changing Electrode Diameter

**Figure 8:**
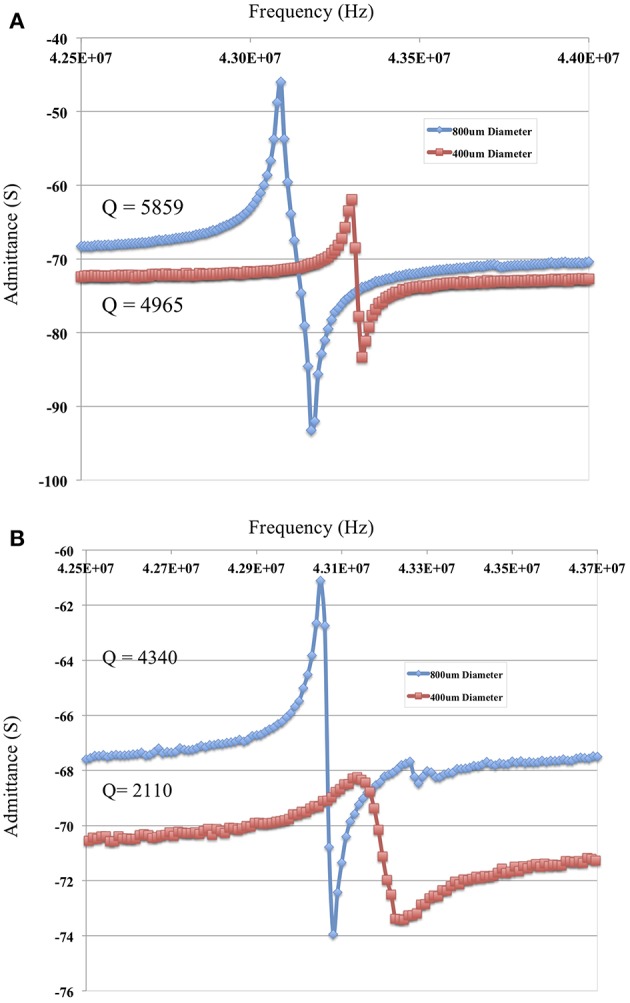
**(A,B)** Increasing electrode diameter improves the *Q*-factor of the micro-resonator. The admittance spectrum of two microresonators with a resonant frequency of approximately 42 MHz and an electrode of 400 μm diameter and a thickness of 33 nm (in red) and a second with a gold electrode of 800 μm diameter and a thickness of 33 nm (in blue) are shown **(A)** in air and **(B)** in fluid.

For bio-sensing applications, electrode size of TSM devices determines the sensing area and impacts sensitivity as demonstrated by Sauerbrey's Equation (1) and subsequently Equation (14). The plot in Figure 4 shows that reducing electrode size allows for designing TSM sensors without inharmonic waves. This relationship is due to the fact that reducing electrode size increases *f*_*e*_ and reduces the range of frequencies that lie between *f*_*e*_ and *f*_*s*_, leading to less inharmonic waves (Figure 1A). On the other hand, as we have discussed in the previous section, increasing electrode resistance (due to reduced electrode size) reduces the *Q*-factor as explained by the modified BVD model. The admittance spectrum of TSM sensors with two different electrode diameters is shown in Figures 8A,B, where the *Q*-factor for the sensor with 800 μm diameter electrode is higher than the sensor with 400 μm diameter. Therefore, reduction in diameter of the electrodes, to increase the spatial resolution of the TSM sensor, reduces the *Q*-factor, which in turn has the effect of decreasing the sensitivity. Simultaneously, the decrease in electrode sensing area (proportional to the square of the diameter) also has the effect of increasing the sensitivity as shown in Equation 14, which compensates for the loss in sensitivity due to lower *Q*-factor.

### 4.5. Cell Adhesion

**Figure 9:**
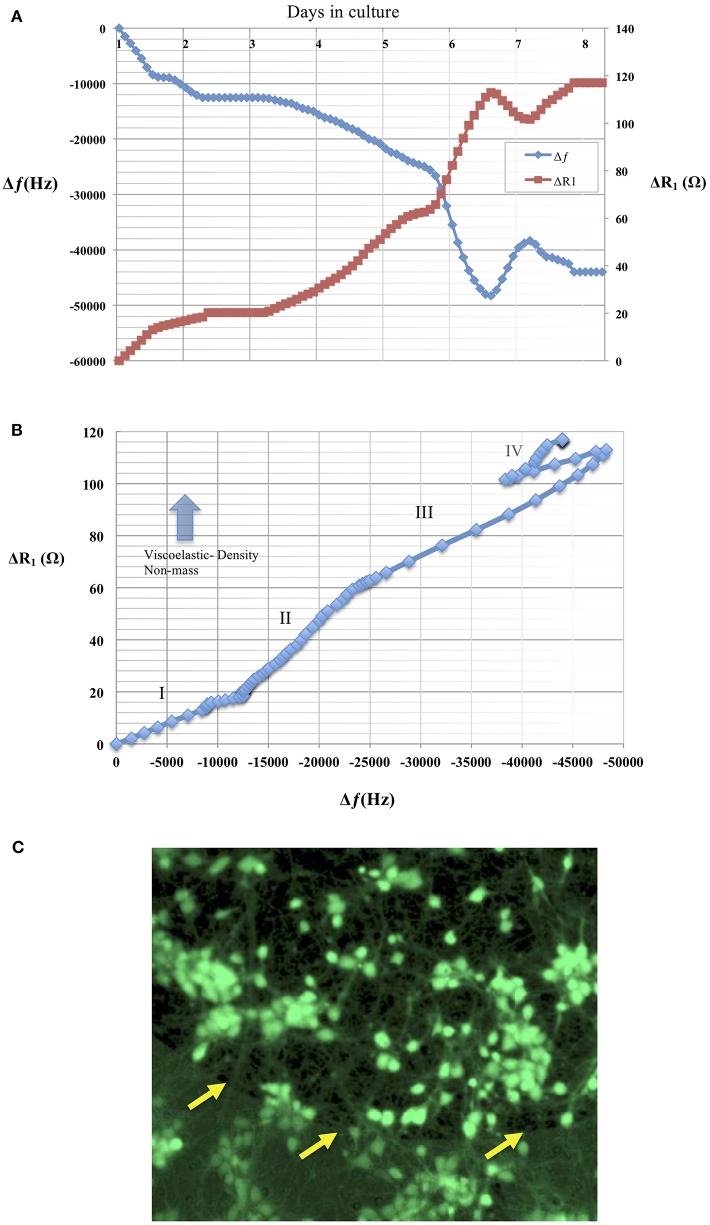
**(A)** Changes in resonant frequency and motional resistance of the TSM sensor are plotted in response to neuronal adhesion over a period of 8 days in culture for a TSM resonator with resonant frequency of 90 MHz and an electrode diameter of 400 μm. **(B)** Plot shows ratio of Δ*R*_1_ vs. Δ*f*_*s*_, Increase in the slope of the curve indicates increase in viscoelastic-density (non-mass) changes in the adhering layer. The plot shows mechanical changes in adhering cell layer over the course of 8 days. Phase I shows a linear behavior indicating both mass and viscoelastic changes in the adhering layer. Phase II shows a slope increase due to increase in the contributing of the viscoelastic changes of the adhering layer. Phase III shows a return to linear behavior and an equal contribution from mass and viscoelastic changes to the curve. Phase IV: shows cell layer exhibits changes in both viscoelastic and mass contributions. **(C)** Live/Dead assay as described of neural culture 7 days after seeding. Live cells are identified by green calcein fluorescence. Arrows in the image are indicating electrode edge.

The advantages offered by TSM sensors that include real-time monitoring of adhering masses have been of interest in the study of mechanics of cell adhesion to artificial substrates. For a long time, the adhesion phenomenon has been investigated using bright field or fluorescence methods that do not easily lend themselves to real-time quantitative assessment of adhesion dynamics. Studies using TSM to track cell adhesion have been extended to a variety of cell lines and pharmacological manipulation of cell cytoskeletal mechanics (Khraiche et al., 2003; Wang and Muthuswamy, 2008). This interest in studying large populations of cells, has even led to the availability of commercial systems to study cell adhesion using TSM sensors. However, the electrode size (in the order of 5–8 millimeters) of commercially available TSM sensors limits these studies to large cell populations (tens or hundreds of thousands of cells). Therefore from Table 1, we chose a sensor prototype that had a large *Q*-factor at the highest resonant frequency (90 MHz) with an electrode diameter of 400 μm (that can accommodate hundreds of cells) that can achieve the highest sensitivity according to Equation (14). Data in Figure 9 shows a mostly monotonic decrease in resonant frequency of a TSM device in response to neuronal adhesion tracked over a period of 8 days. The average standard deviation in the resonant frequencies of these TSM sensors in liquid over a 5-h period, before the addition of neurons, was 31 Hz, demonstrating long-term stability of the TSM sensors in liquid. In addition, the mechanical properties of the adhering cell layer can be tracked in real-time by considering changes in the ratio of Δ*R*_1_ to Δ*f*_*s*_ (Silva and Khraiche, 2013). The data in Figure 9B show a plot of Δ*R*_1_ vs Δ*f*_*s*_ where an increase in the slope of the curve indicates non-mass changes such as viscoelastic density in the adhering layer. The adhesion process typically involves dramatic changes in the cytoskeleton that translates to changes in cell shape, mechanical properties, and cell spreading. These changes are typically difficult to monitor and quantify in real-time using conventional techniques, but it has been shown that TSM sensors are very effective in monitoring such mechanical changes in cells. Additionally, their ability to differentiate between adhering and non-adhering mass makes them ideal for antigen-based cell capture (Khraiche et al., 2005). This performance characteristic is due to the affective lateral sensing layer that extends only a few 100 nm into the solution, which enables TSM sensors to monitor adhered thin films, without being affected by the rest of the medium. This feature offers an advantage when using TSM sensors compared to competing technologies such as EIS (electrochemical impedance spectrum) that typically monitors cell adhesion via a current flow between two neighboring electrodes which can be susceptible to changes in conductivity of the medium and non-adhering cells. In comparison with conventional, commercial TSM sensors, whose electrode diameters are 5–8 mm and a resonant frequency of 5–10 MHz, the electrode diameters of the microscale TSM resonators reported in this current study are at least an order of magnitude smaller, with resonant frequencies at least an order of magnitude larger. Data in Table 1 shows the impact of sensor design parameters on sensitivity as represented by the smallest detectable mass calculated in column 1. The smaller electrode diameters will result in a proportionate decrease in the number of cells on the electrode surface. In principle, an order of magnitude increase in resonant frequency will translate into an increase in sensitivity by two orders of magnitude since the sensitivity is proportional to the square of the resonant frequency. Furthermore, an order of magnitude decrease in the electrode diameter translates into two orders of magnitude increase in sensitivity, since sensitivity is inversely proportional to the square of the electrode diameters.

**Table 1:** Summary of prototypes designs and their corresponding *Q*-factors.

**Δm (ng/mm2)**	**Freq (MHz)**	**Diameter**	**Electrode thickness (nm)**	**Q-factor**
0.54	42	1.2 mm	33	7,020
0.49	42	1.2 mm	230	7,620
0.22	42	800 μm	230	7,600
0.35	42	800 μm	33	4,800
0.073	42	400 μm	230	5,695
0.084	42	400 μm	33	4,965
0.081	50	400 μm	33	4,340
0.023	50	200 μm	230	3,800
0.016	50	150 μm	230	3,000
0.182	77	800 μm	230	5,000
0.049	90	400 μm	230	4,000
0.13	90	800 μm	230	6,000
70.13	10	8 mm	230	10,000

### 4.6. Neuron Adhesion to SWCNT Coated TSMs

**Figure 10:**
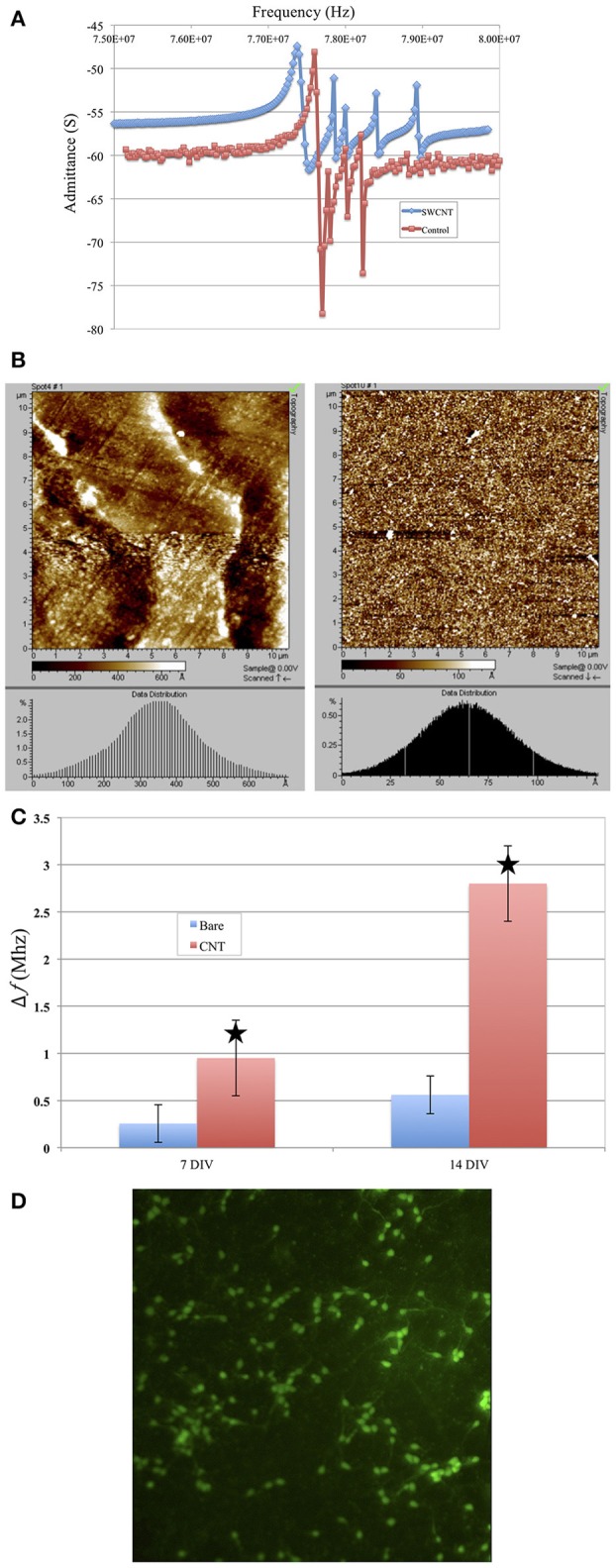
**(A)** Coating top gold electrode with SWCNT reduced the *Q*-factor. Plot in red shows the admittance spectrum before and plot in blue shows admittance spectrum after SWCNT coating. **(B)** Results from AFM scanning of the surface of the coated TSM (Right) electrode vs. the noncoated surface (left). The histogram shows an average surface roughness of 35–40 nm for SWCT coated electrodes vs. 6–7 nm for noncoated. **(C)** Change in resonant frequency of TSM devices after cells are added to sensor surface. Bars in red indicated frequency change due to cells added to TSM electrodes coated with SWCNT. Blue bars indicate frequency due to uncoated (control) TSM surfaces. Overall, the SWCNT sensors show higher resonant frequency drop for cells at similar seeding densities compared to the control (uncoated) surfaces. The star shows the predicted change in resonant frequency for higher surface roughness electrodes. **(D)** Live/dead assay of neurons at day 7 grown on SWCNT.

**Figure 11:**
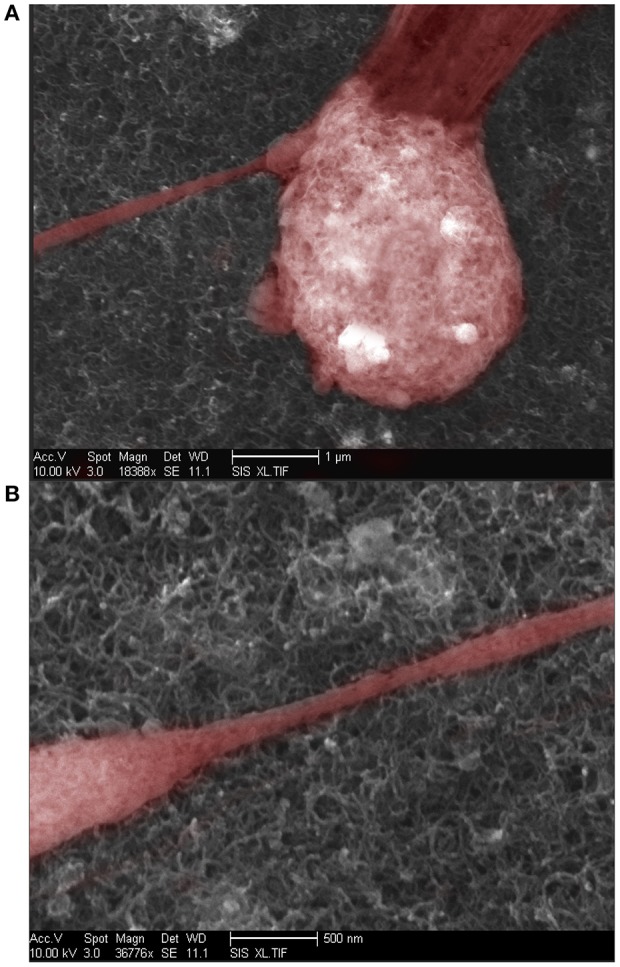
**(A)** Scanning Electron Microscope image of neuron growing over a SWCNT coated surface. **(B)** Scanning electron microscope image of neural processes growing over a SWCNT coated surface.

We investigated the effect of surface roughness on the performance of TSM electrodes by coating the gold electrodes on the quartz with SWCNT (single walled carbon nanotube). The choice of SWCNT is due to their excellent properties for sensing, including high conductivity and high surface to volume ratio. Additionally, nanotopography of SWCNT is of great interest for studying cell adhesion to nano-substrates (McHale and Newton, 2004; Ballerini, 2008; Khraiche et al., 2009; Hecht et al., 2011). We coated the gold electrodes with SWCNT via drop-casting which resulted in almost 4-fold increase in surface roughness measured via AFM (Figure 10B). Results in Figure 10A shows the *Q*-factor is reduced for the TSM device coated with SWCNT. Acoustic losses reduce the *Q*-factor due to defects, scattering and losses at the acoustic boundaries (Figure 10A) (Heitmann and Wegener, 2007). In addition, the presence of high surface roughness leads to slip conditions between the solid and liquid phases as the TSM sensor is operated in liquid, leading to an increase in Δ*f*. Furthermore, TSM sensors have been shown to be capable of tracking cell adhesion quantitatively in real-time under various chemical and surface treatments (Mindlin and Deresiewicz, 1954; Khraiche et al., 2005; Sapper et al., 2006; Khraiche and Muthuswamy, 2012). In the final section of this work, we investigated TSM sensor response (change in resonant frequency) to cell adhesion on SWCNT modified electrodes over a period of 14 days. Measurement time points were chosen to collect data well past the typical maturity of disassociated neurons, as indicated by an advanced state of axonal growth and spreading. This experiment allowed us to quantify the adhesion response of neurons on gold vs. SWCNT. The data in Figure 10C shows that TSM sensors coated with SWCNT have a significantly larger change in resonant frequency to the same density of adhering cells at two time points (7 and 14 days *in vitro*). The increase in TSM response correlates with observations of enhanced adhesion of neurons on CNTs and was consistent with the predicted Δ*f* in Equation 16 (Figure 10D). Scanning electron micrographs (SEM) in Figure 11 show adhesion of individual processes of neurons grown on SWCNT.

## 5. Conclusion

Thickness shear mode piezoelectric sensors have shown great promise in bio-sensing applications but currently fall short in the sensitivity and detection area as compared to competing sensing modalities such as surface plasmon resonance (SPR). This report highlights key design principles for improving sensitivity and lowering the detection area for TSM sensors operating in liquids, for the purposes of monitoring cell adhesion in real time. The theoretical predictions have been validated with fabricated prototypes operating in liquid. The plate-back equation first derived by Mindlin et al. was used to eliminate unwanted inharmonic standing waves that interfere with the correct prediction of the sensor's resonant frequency (Mindlin and Deresiewicz, 1954). We added a new term to include the effect of liquid and higher density and viscosity coatings on inharmonic suppression which changes the design space previously suggested for inharmonic suppression. We also used the prototypes with the highest sensitivity and smallest sensing area to monitor neuronal adhesion. In addition, we used TSM sensors to probe cell responses to SWCNT. Finally, reducing the size of the sensing area to a 150–400 μm for TSM devices, improves the spatial resolution by monitoring 100–1,000s of neurons. This technology remains an important tool in studying cell adhesion as they provide real-time, label-free information and hold many advantages over competing technologies such as EIS for monitoring cell adhesion. The theoretical guidelines in this work lay out the interplay among sensor design parameters, such as sensing area, frequency of the quartz, and thickness of the electrode, and how they affect sensor performance. The design of TSM sensors should be application driven so as to set the expectation of one or more aspects of the design specification while tuning the rest of the parameters to produce the desired performance.

## Data Availability

The datasets generated for this study are available on request to the corresponding author.

## Author Contributions

MK, JR, and JM contributed to the conceptualization and design of the TSM sensors. MK fabricated and tested the TSM sensor prototypes and performed the *in vitro* and bench top experiments. MK and JM also contributed to the experimental design and writing of the manuscript.

### Conflict of Interest Statement

The authors declare that the research was conducted in the absence of any commercial or financial relationships that could be construed as a potential conflict of interest.
